# The National COVID Cohort Collaborative: Analyses of Original and Computationally Derived Electronic Health Record Data

**DOI:** 10.2196/30697

**Published:** 2021-10-04

**Authors:** Randi Foraker, Aixia Guo, Jason Thomas, Noa Zamstein, Philip RO Payne, Adam Wilcox, N3C Collaborative

**Affiliations:** 1 Division of General Medical Sciences School of Medicine Washington University in St. Louis St. Louis, MO United States; 2 Institute for Informatics School of Medicine Washington University in St. Louis St. Louis, MO United States; 3 Department of Biomedical and Medical Education School of Medicine University of Washington Seattle, WA United States; 4 MDClone Ltd Beer Sheva Israel; 5 See Acknowlegments

**Keywords:** synthetic data, protected health information, COVID-19, electronic health records and systems, data analysis

## Abstract

**Background:**

Computationally derived (“synthetic”) data can enable the creation and analysis of clinical, laboratory, and diagnostic data as if they were the original electronic health record data. Synthetic data can support data sharing to answer critical research questions to address the COVID-19 pandemic.

**Objective:**

We aim to compare the results from analyses of synthetic data to those from original data and assess the strengths and limitations of leveraging computationally derived data for research purposes.

**Methods:**

We used the National COVID Cohort Collaborative’s instance of MDClone, a big data platform with data-synthesizing capabilities (MDClone Ltd). We downloaded electronic health record data from 34 National COVID Cohort Collaborative institutional partners and tested three use cases, including (1) exploring the distributions of key features of the COVID-19–positive cohort; (2) training and testing predictive models for assessing the risk of admission among these patients; and (3) determining geospatial and temporal COVID-19–related measures and outcomes, and constructing their epidemic curves. We compared the results from synthetic data to those from original data using traditional statistics, machine learning approaches, and temporal and spatial representations of the data.

**Results:**

For each use case, the results of the synthetic data analyses successfully mimicked those of the original data such that the distributions of the data were similar and the predictive models demonstrated comparable performance. Although the synthetic and original data yielded overall nearly the same results, there were exceptions that included an odds ratio on either side of the null in multivariable analyses (0.97 vs 1.01) and differences in the magnitude of epidemic curves constructed for zip codes with low population counts.

**Conclusions:**

This paper presents the results of each use case and outlines key considerations for the use of synthetic data, examining their role in collaborative research for faster insights.

## Introduction

COVID-19 presents data and knowledge sharing challenges [1]. Clinical data exist at individual institutions; however, these data are rarely shared with external entities. Big data from multiple institutions allow for more comprehensive analyses, particularly for characterizing rare outcomes [2,3]. In response to this need, the National COVID Cohort Collaborative (N3C), an open science community, was formed to ingest and harmonize COVID-19 data from institutions across the United States [4]. The N3C sought a solution to preserve the privacy and confidentiality of these clinical data while enabling their broad dissemination [5-7] and partnered with MDClone (Beer Sheva, Israel) to computationally derive “synthetic” N3C data and support the rapid advancement of population health insights [8].

Since synthetic derivatives of data can enable privacy-preserving data downloads and accelerate discovery, these data assets can potentially be of great utility to the N3C and the broader informatics community. Our synthetic data validation workstream was established to assist the N3C community in better understanding the utility of synthetic data for research purposes. Our previous work demonstrated statistical equivalency between original and computationally derived data sets from a local instance of MDClone [9]. We also used synthetic data sets exclusively to apply machine learning to predict decompensation in heart failure [10]. Others have demonstrated repeatedly generated synthetic data sets from MDClone produced stable results that were similar to the original data [11].

However, the performance of MDClone—the comparison of original to synthetic data—has not been validated using data comprising multiple sources such as those originating across health systems. To that end, we tested three use cases, including (1) exploring the distributions of key features of the COVID-19–positive cohort; (2) training and testing predictive models for assessing the risk of admission among these patients; and (3) determining geospatial and temporal COVID-19–related measures and outcomes, and constructing their epidemic curves. We analyzed data for each use case using original and synthetic data. We conducted analyses using traditional statistics, machine learning approaches, and temporal and spatial representations of the data. Here we present the results of these analyses and describe the strengths and limitations of using synthetic data for research.

## Methods

### Overview

The Clinical and Translational Science Award Program developed N3C in response to a need for integrating, harmonizing, and democratizing individual-level COVID-19 data [4]. The N3C established a secure data enclave to store data and conduct collaborative analytics. The subsequent analyses resulted from a synthetic data pilot designed to evaluate the utility of computationally derived data for the N3C community. Synthetic data generation represents an emerging technology that can support population health research at scale.

As described in more detail elsewhere [9], MDClone uses a computational derivation approach. Briefly, novel data whose features are queried independently for each distinct use case are produced in a multidimensional space that adheres to the statistical properties of the original source data. MDClone censors categorical values that are unique to few patients by removing the value and replacing it with the word “censored” in the computationally derived data set. Extreme numerical values also do not appear in the synthetic data set. Together, these approaches ensure that outliers in the original data set will not be identifiable in the synthetic data derivative.

All analyses were conducted using original data and computationally derived data, respectively, which allowed us to compare the results of analyses and assess the strengths and limitations of leveraging synthetic data for COVID-19 insights. All statistical analyses on the original and synthetic data sets were done outside of MDClone on the Palantir Foundry Analytic Platform (Palantir Technologies).

Analyses were conducted using Python (3.6.10l Python Software Foundation). We obtained institutional review board approval from our institutions for these analyses, in addition to completing data use agreements and requests with the National Center for Advancing Translational Sciences at the National Institutes of Health.

### Use Case 1: Exploring the Distributions of Key Features of the COVID-19–Positive Cohort

The goal of this use case was to evaluate whether synthetic data had similar distributions of demographic and clinical characteristics among the COVID-19–positive cohort as compared to original data. Key characteristics (n=15) of the COVID-19–positive cohort were extracted from MDClone to compare distributions between the synthetic and the original data. The 15 features included age, gender, race, patients’ state of residence, institution, median household income, BMI, number of days between testing positive and hospital admission (if hospitalized), diagnosis of diabetes, dyspnea, chronic kidney disease (CKD), fever, cough, and in-hospital mortality. We calculated mean and SD for continuous variables, and counts and proportions for categorical variables.

### Use Case 2: Training and Testing Predictive Models for Assessing the Risk of Admission Among COVID-19–Positive Patients

The goal of this use case was to evaluate whether synthetic data would perform similarly when training and testing predictive machine learning models on synthetic data as compared to training and testing the models on original data. We included 230,703 patients who tested positive for COVID-19. Features for predictive modeling included 11 variables: age, gender, race, median household income, BMI, minimum oxygen saturation, diabetes, dyspnea, CKD, fever, and cough. These variables were chosen because of initial data suggesting their significant impact on COVID-19 outcomes.

We calculated odds ratios (ORs) and 95% CIs for admission within 14 days of a COVID-19 diagnosis by univariate logistic regression (LR) and multivariable LR using synthetic and original data, respectively. We then developed two widely used machine learning models, random forest (RF) and LR, to predict admission within 14 days of a COVID-19 diagnosis based on the 11 features. We randomly split the cohort into training (80%) and testing (20%) data. The models were trained on the 80% subset of the data and then tested on the remaining 20%. We used a variety of metrics, including accuracy, precision, recall, F1-score, area under the receiver operating characteristic (ROC) curve, and precision-recall curves to evaluate model performance. Each model was trained and evaluated on the synthetic data set, the results of which were then compared against a model trained and evaluated on the original data.

### Use Case 3: Determining Geospatial and Temporal COVID-19–Related Measures and Outcomes, and Constructing Their Epidemiologic Curves

The purpose of this analysis was to assess concordance of geospatial and temporal relationships between the synthetic and original data to make the data actionable and interpretable according to geography and time. Our data sets (original: n=1,854,968 tests; synthetic: n=1,854,950 tests) were event-based with each row representing a patient’s first COVID-19 test result. The data sets included the following variables: source partner with which the patient was affiliated; lab test result (negative/positive); lab test date and time (reference time point for data generation); age at confirmed lab test result; admission start date (days from reference if admission occurred within ±7 days of COVID-19–positive test result); admission length of stay (in days); death (yes/null) during admission; patient’s state of residence; patient’s 5-digit zip code; and median household income, percent of residents under the poverty line, percent without health insurance, and total population by zip code.

On both the synthetic and original data sets, we calculated the aggregate count, 7-day midpoint moving average, and 7-day slope (count – count 6 days prior) per day for positive tests. We then plotted epidemic curves (Plotly version 4.14.1, Plotly Technologies Inc) for positive tests with synthetic and original data overlaid in the same figure. To test for significant differences or equivalence between the synthetic and original data epidemic curves, the paired two-sided *t* test (scipy version 1.5.3, stats.ttest_rel) and two-sided Wilcoxon signed rank test (scipy version 1.5.3, stats.wilcoxon) were run for each metric (count, 7-day moving average, and 7-day slope) treating the counts for individual dates as pairs.

Next, we calculated the differences in the mean, SD, median, IQR, and missingness of zip code–level social determinants of health (SDOH) variables within the original data set. We then compared these original data SDOH values for unique zip codes in the original data that were censored versus uncensored in the synthetic data. We defined censored zip codes as those present within the original data set that could not be matched (n=11,222) within the synthetic data set either due to not being present or being labeled as *censored* within the synthetic data set. We defined uncensored zip codes as present within both the synthetic data and original data (n=5819).

### Ethics

This study was reviewed and approved by the Washington University in St. Louis’ and the University of Washington’s institutional review boards.

## Results

### Use Case 1: Exploring the Distributions of Key Features of the COVID-19–Positive Cohort

The MDClone synthetic data process generated 230,650 participants, compared to 230,703 in the original data. Demographic and clinical variables comparing synthetic and original data sets are displayed in Table 1. The mean age from both data sources was the same (mean 41.6, SD 20.4 years; Table 1). Approximately 47% of patients were male and 53% were White in both data sources. The values of all means and SDs (or counts and percentages) were the same or very similar between original and synthetic data. Table 1 shows that the distribution of demographic and clinical variables was similar between original and synthetic populations.

**Table 1:** Comparison of patient characteristics of available demographic and clinical variables: original vs synthetic data.

		Original data (n=230,703)	Synthetic data (n=230,650)
Age (years), mean (SD)		41.6 (20.4)	41.6 (20.4)
Gender (male), n (%)		108,194 (46.9)	107,892 (46.8)
**Race, n (%)**			
	White	121,706 (52.8)	121,564 (52.7)
	Black	40,930 (17.7)	40,824 (17.7)
	Asian	5203 (2.3)	5117 (2.2)
	Other/unknown	62,864 (27.2)	62,733 (27.2)
**Top 5 most prevalent states, n (%)**			
	1	29,875 (12.9)	28,617 (12.4)
	2	21,191 (9.2)	20,671 (9.0)
	3	21,045 (9.1)	20,319 (9.0)
	4	18,006 (7.8)	16,998 (7.4)
	5	14,391 (6.2)	13,840 (6.0)
**Top 5 most prevalent institutions, n (%)**			
	1	33,413 (14.5)	32,743 (14.2)
	2	24,533 (10.6)	23,986 (10.4)
	3	15,578 (6.8)	15,065 (6.5)
	4	11,870 (5.1)	11,255 (4.9)
	5	11,354 (4.9)	10,850 (4.7)
Household income (US $), median (IQR)		56,738 (45,214, 71,250)	56,662 (45,223, 71,029)
BMI, mean (SD)		30.3 (8.4)	30.3 (8.2)
Admission start date (days from reference), mean (SD)		2.1 (3.3)	2.0 (3.2)
Minimum oxygen saturation, mean (SD)		90.9 (10.1)	91.0 (9.7)
Diabetes, n (%)		31,942 (13.8)	31,929 (13.8)
Dyspnea, n (%)		20,867 (9.0)	20,826 (9.0)
Chronic kidney disease, n (%)		11,225 (4.9)	11,194 (4.9)
Fever, n (%)		30,210 (13.1)	30,200 (13.1)
Cough, n (%)		39,703 (17.2)	39,689 (17.2)
Deceased, n (%)		1133 (0.5)	1008 (0.4)

### Use Case 2: Training and Testing Predictive Models for Assessing the Risk of Admission Among COVID-19–Positive Patients

Features (n=11) used for prediction included age, gender, race, median household income, BMI, minimum oxygen saturation, diagnosis of diabetes, dyspnea, CKD, fever, and cough. Table 2 shows the OR for admission and for each of the 11 variables by univariable LR yielded by original and synthetic data sources, respectively. The comparison of ORs between original and synthetic data sources show that the values for all 11 features were the same or similar. For example, the OR for admission by age from the original data was 1.04 (95% CI 1.04-1.04), which was the same as that obtained from synthetic data.

**Table 2:** Logistic regression for admission: original vs synthetic data.

	Univariate LR^a^, OR^b^ (95% CI)		Multivariable LR, OR (95% CI)	
	Original data	Synthetic data	Original data	Synthetic data
Age	1.04 (1.04-1.04)	1.04 (1.04-1.04)	1.00 (1.00-1.00)	1.00 (1.00-1.00)
Male gender	1.20 (1.16-1.24)	1.14 (1.10-1.17)	1.11 (0.99-1.23)	1.03 (0.93-1.15)
Black race	2.15 (2.07-2.22)	2.09 (2.02-2.17)	0.99 (0.87-1.12)	0.93 (0.82-1.06)
Median household income	1.00 (1.00-1.00)	1.00 (1.00-1.00)	1.00 (1.00-1.00)	1.00 (1.00-1.00)
BMI	1.02 (1.01-1.02)	1.02 (1.01-1.02)	0.97 (0.97-0.98)	1.01 (1.00-1.02)
Minimum oxygen saturation	0.97 (0.96-0.97)	0.97 (0.96-0.97)	0.97 (0.97-0.98)	0.97 (0.97-0.98)
Diabetes	6.14 (5.94-6.34)	6.15 (5.95-6.36)	1.45 (1.29-1.62)	1.46 (1.30-1.63)
Dyspnea	4.79 (4.62-4.97)	4.79 (4.61-4.97)	1.23 (1.09-1.38)	1.25 (1.11-1.41)
Chronic kidney disease	7.20 (6.89-7.52)	7.17 (6.87-7.49)	1.23 (1.07-1.42)	1.26 (1.09-1.45)
Fever	2.62 (2.52-2.71)	2.62 (2.53-2.72)	1.44 (1.29-1.61)	1.45 (1.30-1.62)
Cough	1.38 (1.33-1.43)	1.38 (1.32-1.43)	1.50 (1.32-1.70)	1.45 (1.28-1.65)

^a^LR: logistic regression.

^b^OR: odds ratio.

The comparison of ORs between original and synthetic data sources shows that the multivariable LR yielded the same or similar results. For example, the OR for admission by Black race from the original data was 0.99 (95% CI 0.87-1.12), which was similar to that obtained from synthetic data (OR 0.93, 95% CI 0.82-1.06). Of note, the ORs that corresponded to a one-unit increase in BMI were on either side of the null (0.97 vs 1.01).

**Figure 1:**
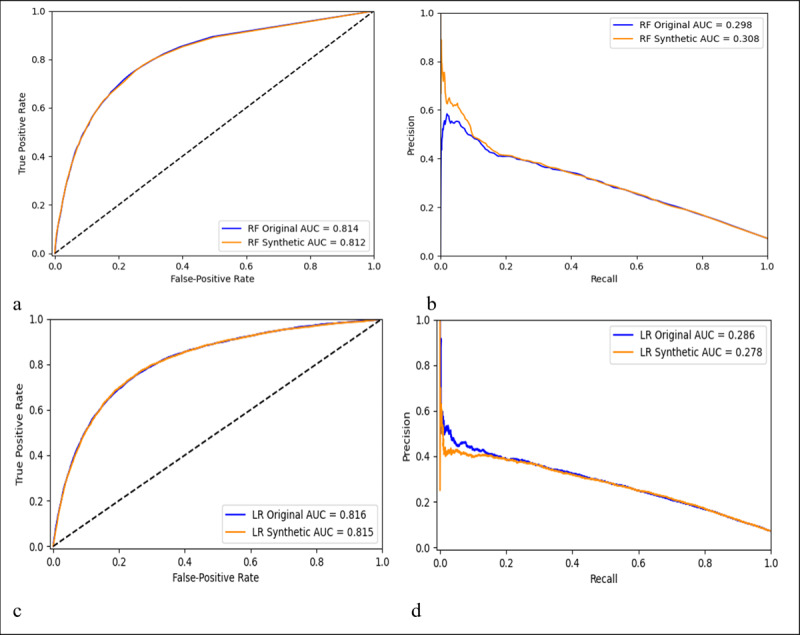
Prediction performance for the two models by receiver operating characteristic curves (A, C) and precision-recall curves (B, D) by using original and synthetic data. Results for the RF model are in the first row (A, B); the second row (C, D) is for LR. AUC: area under the curve; LR: logistic regression; RF: random forest.

The machine learning models that were trained and tested on original data and then trained and tested on synthetic data used the same 11 features. Figure 1 shows the comparison of model prediction performance using original and synthetic data, respectively. We found the RF model achieved an under the ROC curve of 0.814 (0.816 by LR) using original data, and 0.812 (0.815 by LR) using synthetic data (Figure 1 A and C). Meanwhile, the RF model achieved an average precision of 0.298 (0.286 by LR) with original data and 0.308 (0.278 by LR) with synthetic data (Figure 1 B and D).

**Figure 2:**
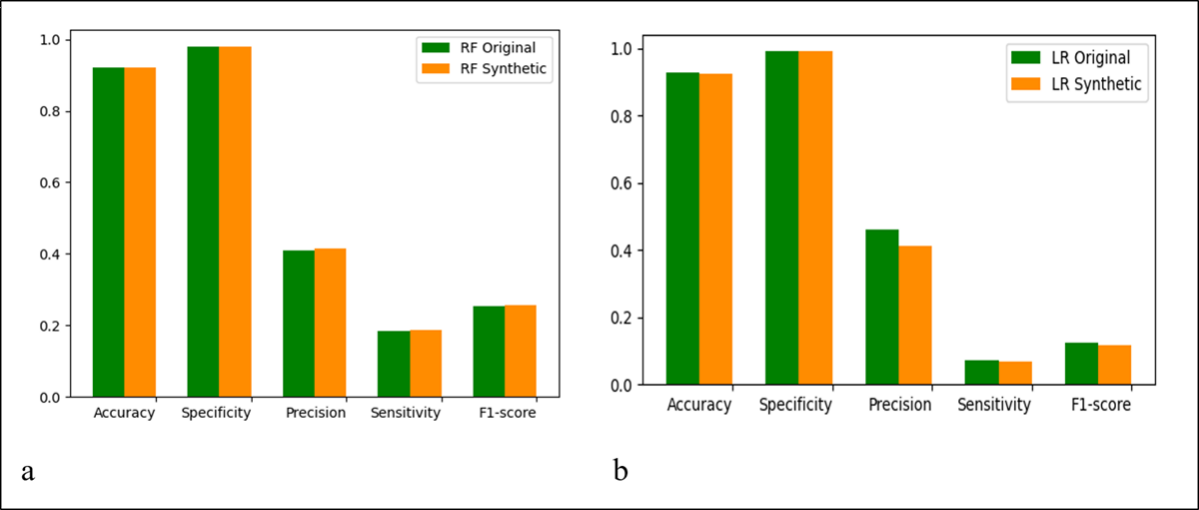
Model performance metrics from original (green) and synthetic (gold) data by accuracy, specificity, precision, sensitivity, and F1-score: RF model (A) and LR model (B). LR: logistic regression; RF: random forest.

Figure 2 shows additional metrics for the evaluation of model performance. We observed the same or similar patterns by accuracy, specificity, precision, sensitivity, and F1-score when comparing models that were trained and tested on original data as compared to those trained and tested on synthetic data.

**Figure 3:**
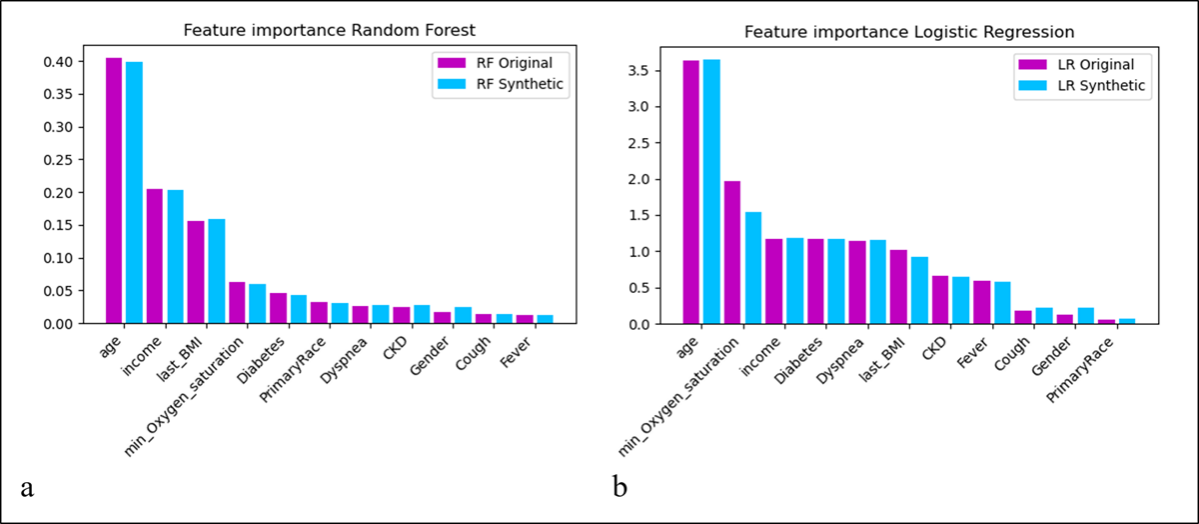
Feature importance for the 11 variables in RF (a) and LR (b) models: original vs synthetic data. CKD: chronic kidney disease; LR: logistic regression; RF: random forest.

Figure 3 shows the feature importance according to RF (Figure 3 A) and LR models (Figure 3 B) using original (magenta) and synthetic (blue) data. Both the RF and LR models’ demonstrated that features such as age, income, and minimum oxygen saturation were high-ranking informative features.

### Use Case 3: Determining the Zip Code–Level Distributions of COVID-19–Related Outcomes and Calculating Their Epidemiologic Curves

**Figure 4:**
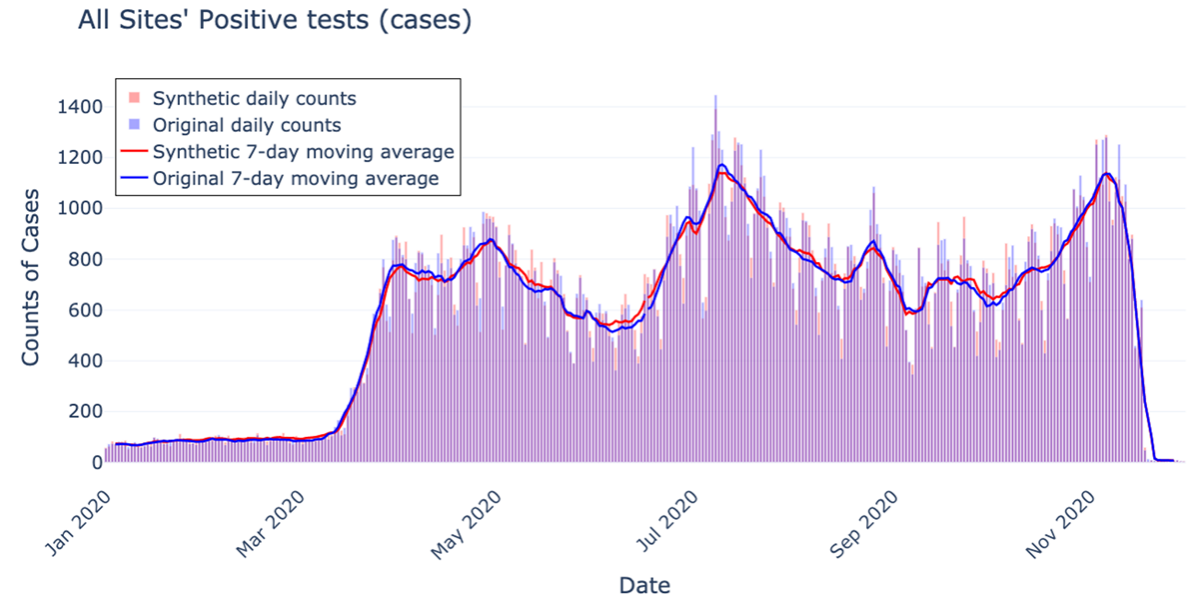
Original data (light blue) and synthetic data (light red), with their overlap (purple).

A graphical comparison of the epidemic curves for aggregate positive tests (cases) between the synthetic and original data is shown in Figure 4. Pairwise statistics for the epidemic curve metrics are shown in Table 3; no significant differences were found between the synthetic and original data epidemic curves across all metrics (Wilcoxon signed rank test *P* value range .50-.90; Student paired *t* test *P* value range .996-.998).

Compared to censored zip codes, uncensored zip codes had a higher median household income, a lower percentage of residents under the poverty line, a lower percentage of patients without health insurance, a higher total population, and fewer missing values for all four SDOH. Total population and data missingness were the two greatest differences between uncensored and censored zip codes. Uncensored zip codes had a 74% higher median total population and had approximately 70% fewer missing SDOH values than censored zip codes (Table 4).

**Table 3:** Epidemic curves aggregate cases’ paired statistical tests, comparing original to synthetic data.

Metric	Date range	Wilcoxon result	Wilcoxon *P* value	*t* statistic	*t* test *P* value
Counts	335	26,288	.50	–0.002	>.99
7-day moving average	329	26,005	.78	–0.006	>.99
7-day slope	329	25,788.5	.90	–0.002	>.99

**Table 4:** SDOH values for zip codes that were uncensored (n=5819) compared to censored (n=11,222) zip codes.

SDOH^a^ and censored status		Mean	SD	Median	IQR	% missing
**Median household income (US $)**						
	Uncensored	63,536	26,755	57,352	28,692	3.28
	Censored	60,544	26,549	54,358	27,067	10.98
	Difference (%)	+2992 (4.9)	+206 (0.8)	+2994 (5.5)	+1625 (6.0)	–7.70 (70.1)
**Percent under poverty line**						
	Uncensored	12.89	8.74	10.80	10.40	2.92
	Censored	13.87	10.15	11.60	11.50	9.12
	Difference (%)	–0.98 (7.1)	–1.41 (13.9)	–0.80 (6.9)	–1.10 (9.6)	–6.20 (68.0)
**Percent without health insurance**						
	Uncensored	8.52	5.09	7.50	6.50	2.84
	Censored	9.65	7.09	8.10	8.00	9.00
	Difference (%)	–1.13 (11.7)	–2.00 (28.2)	–0.60 (7.4)	–1.50 (18.8)	–6.16 (68.4)
**Total population**						
	Uncensored	17,363	16,128	12,263	23,172	2.73
	Censored	14,540	17,317	7048	21,436	8.69
	Difference (%)	+2823 (19.4)	–1189 (6.9)	+5215 (74.0)	+1736 (8.1)	–5.96 (68.6)

^a^SDOH: social determinants of health.

## Discussion

### Principal Findings

Our main findings demonstrated that computationally derived data had the same or similar statistical output as the original data sets, with the caveat that zip codes with a lower population had data suppressed/censored for privacy reasons more often than zip codes with a higher population. In each use case, the results of the analyses appear sufficiently similar between the synthetic derivative and the original data across the various methods used to assess similarity (means, medians, *P*>.05, overlapping CI, etc) to draw the same conclusions with the exception of one OR on either side of the null in multivariable analyses (0.97 vs 1.01). In several instances, the results were exactly the same and rarely were there statistically significant differences between data sets.

Small sample sizes, missing values, and high dimensionality can all adversely affect the data synthesis process and the precision and interpretability of original data. Our geospatial analysis shows that zip codes that are censored to protect patient privacy have a lower population, which will likely make using these computationally derived data to study rural populations more challenging. Additionally, the lower original data quality found within censored zip codes—seen in greater SDOH missingness—as compared to uncensored may indicate broader data quality issues in rural zip code data. Such issues may pose a further challenge to data synthesis.

This was the first validation of computationally derived data using the N3C data. Our study adds to the growing literature of synthetic data validation in the following ways. First, our study is the first assessment of N3C synthetic data utility and has been conducted prior to the broad dissemination of N3C synthetic data. Thus, our study provides insight to the validity of N3C synthetic data prior to its dissemination for use by the broader N3C community. Second, our results from use case 3 support the temporal validity of these computationally derived data as an alternative to date-shifting when privacy must be protected yet temporality maintained.

For these descriptive and quantitative analyses, the synthetic data appear to produce similar patterns and results compared to the original data, except for in the context of high missingness. We acknowledge that these use cases may not represent all possible ways in which the synthetic data may be used by the N3C community and thus validation should continue. In addition to continuously validating these data for different use cases and analytic methods, we seek to explore the performance of other commercial systems in the N3C community and their approaches to synthetic data generation and the privacy-preserving aspects of each approach.

We also suggest that the synthetic data can be used by researchers for hypothesis generation to then be validated later on original data. Another potential use case that could be valuable to the N3C community, which we do not explicitly test here, is the potential for synthetic data to be used for software engineering projects that seek to develop digital health tools for combating the COVID-19 pandemic. Computationally derived data that are faithful to the original data could be used to develop and test such tools.

### Limitations

For these analyses, we compared the data statistically and did not conduct privacy evaluations of the synthetic data that will be a focus of future investigations. We used a *P* value threshold of .05 to maintain simplicity of presenting results from multiple use cases. We acknowledge that such thresholds would (and should) vary by use case and specifically by the amount of error a researcher is willing to tolerate given the context of the research question. We also acknowledge that other statistical tests such as equivalence testing could be suitable to assess the equivalence of computationally derived data to original data. However, the threshold for equivalence will yet again depend on the use case.

Our geospatial and temporal analysis was limited in scope. Our work is ongoing, and future analyses will assess validity of other measures (eg, tests, admissions, deaths, or positivity) over time—both in aggregate and at the zip code level—in greater detail.

### Conclusions

We conclude that the potential for leveraging synthetic data for the conduct of COVID-19 research in N3C is substantial. We expect that the use of synthetic data will accelerate the conduct of data-driven research studies across the community, as it will allow the N3C to overcome data sharing barriers and rapidly create COVID-19 analytic insights [4]. Future directions for this work include developing and validating additional clinical risk prediction models, using a larger repertoire of analytic methods, conducting geospatial and temporal analyses in greater detail and at the zip code level, and evaluating additional strengths and limitations of computationally derived data for research [1].

### Clinical Relevance Statement

Data synthesis platforms like MDClone are expected to enhance the N3C community’s ability to use clinical data for faster COVID-19 insights and reduce barriers to data access by multiple stakeholders.

## Abbreviations

CKD: chronic kidney disease
ITM: Institute for Translational Medicine
LR: logistic regression
NCATS: National Center for Advancing Translational Sciences
NIH: National Institutes of Health
N3C: National COVID Cohort Collaborative
OR: odds ratio
RF: random forest
ROC: receiver operating characteristic
SDOH: social determinants of health
